# Network analysis of trauma-related mental health comorbidities under continuous traumatic stress: a comparative study of South African and Ukrainian adolescents

**DOI:** 10.3389/fpsyt.2026.1888773

**Published:** 2026-10-13

**Authors:** Larysa Zasiekina, Alberta S. J. van der Watt, Mae Holland, Olena Solonenko, Soraya Seedat

**Affiliations:** 1 Department of Psychology, University of Exeter, Exeter, United Kingdom; 2 Department of General and Clinical Psychology, Lesya Ukrainka Volyn National University, Lutsk, Ukraine; 3 Department of Psychiatry, Faculty of Medicine and Health Sciences, Stellenbosch University, Tygerberg, South Africa; 4 South African Medical Research Council Genomics of Brain Disorders Research Unit, Department of Psychiatry, Faculty of Medicine and Health Sciences, Stellenbosch University, Tygerberg, South Africa

**Keywords:** adolescents, chronic violence exposure, continuous traumatic stress (CTS), cross-cultural comparison, network analysis, posttraumatic stress disorder (PTSD), transdiagnostic symptoms

## Abstract

**Background:**

Adolescents living under conditions of continuous traumatic stress (CTS) are exposed to ongoing, persistent, unpredictable threats that differ from past discrete traumatic events and are associated with high levels of CTS symptoms, posttraumatic stress disorder (PTSD), depression, and anxiety. Despite growing evidence that CTS functions as a risk factor for trauma-related disorders, little is known about how specific CTS symptoms are embedded within broader psychopathology networks across diverse contexts of chronic violence.

**Aim:**

This study applied item-level network analysis to examine the structural role of individual CTS symptoms within PTSD, depression, and anxiety symptom networks among adolescents exposed to CTS in South Africa (*n* = 600) and Ukraine (*n* = 485).

**Method:**

Adolescents (aged 12 to 18 years) completed standardised measures of CTS, PTSD, depression, and anxiety symptoms. Item-level network models were used to examine within- and cross-domain symptom associations, identify central and bridging CTS symptoms, assess their predictive value for overall symptom severity, and test network invariance across countries.

**Results:**

In both samples, strong within-domain clustering was observed alongside robust cross-domain connections linking CTS symptoms with PTSD, depression, and anxiety symptoms. In Ukraine, CTS symptoms were represented by symptoms reflecting loss of meaning, intense fear or horror, and episodes of rage, while in South Africa, CTS symptoms were represented by difficulty controlling emotions, feelings of betrayal, and perceived life threat. The findings also suggest that CTS symptoms were more tightly interconnected and more evenly distributed across PTSD, depression, and anxiety symptoms in the Ukrainian sample, whereas the South African sample demonstrated a broader and more diffuse symptom pattern, with anxiety-related symptoms appearing to be more prominently expressed.

**Conclusion:**

Findings suggest that CTS symptoms may potentially operate as a transdiagnostic process – cutting across, and contributing to shared variance in PTSD, depression, and anxiety symptoms – among adolescents exposed to ongoing violence. Interventions targeting core CTS bridge symptoms may reduce comorbidity and improve mental health outcomes in chronically threatened youth across diverse conflict settings.

## Introduction

Populations living under conditions of continuous exposure to violence experience substantial psychological distress while attempting to survive in unsafe, unpredictable environments (1–3). Such conditions are widespread globally and include contexts of ongoing armed conflict and community violence, both of which expose individuals to sustained current and future threat rather than discrete traumatic events, representing a major public mental health challenge (4).

A prominent example of war-related continuous violence is the ongoing Russia-Ukraine war, beginning with the full-scale invasion of Ukraine in February 2022. This conflict has been formally recognised as a humanitarian crisis, with over 13,300 civilian deaths reported to date (5). Continuous exposure to warfare, displacement, and associated losses has had profound consequences for the mental health of those living in, or displaced from, the region (6–9).

A parallel example of chronic violence is observed in South Africa, where endemic crime represents a persistent threat to personal safety, especially for women and youth (10, 11). South Africa has among the highest global rates of homicide and femicide, averaging 5.5 femicide cases in every 100,000 women; nearly five times the global average for intimate partner violence (1.3 cases for every100,000 women) (12). Violent crimes such as armed robbery and contract killings remain pervasive and deeply rooted in the country’s apartheid legacy and ongoing structural inequality (13, 14), with exposure often occurring not only at the individual level but also through repeated witnessing and shared experience of violence within communities, and resulting in enduring consequences for population-level mental health and well-being (15, 16).

In contexts of chronic violence, access to systems of support is limited or unavailable, affecting not only service users of health and social care, but also mental health professionals themselves (17). Burnout, secondary traumatic stress, and compassion fatigue among healthcare professionals impact the quality and effectiveness of care and psychological support (17–19).

Where psychological interventions are available, they are typically framed within a posttraumatic stress disorder (PTSD) paradigm. PTSD frameworks, screening tools, and diagnostic criteria, based on traumatic events that have passed, remain dominant in both research and clinical practice (20, 21). According to the Diagnostic and Statistical Manual version 5 (DSM-5), PTSD symptoms are characterized by four clusters, including intrusion, avoidance, negative alterations in cognition and mood, and arousal/reactivity (22). Similarly, the International Classification of Diseases version 11 (ICD-11) conceptualises PTSD through re-experiencing, avoidance, and a persistent sense of current threat (23). Crucially, both diagnostic systems assume that trauma is located in the past, and that symptoms emerge after the traumatic event has ended. This assumption has been increasingly questioned in contexts of ongoing, inescapable threat, where danger has not ceased, and the future remains unpredictable.

Also, first-line evidence-based protocols for treating PTSD, such as cognitive therapy and cognitive behavioural therapy, typically involve processing a traumatic memory as a past, time-limited event. These approaches to memory updating often use “bookends” to anchor the beginning and end of the trauma, helping the client recognise that the threat is over and that they are now relatively safe (24). They also include techniques aimed at modifying maladaptive beliefs and re-evaluating perceived threat. However, these strategies may be less suitable in contexts of ongoing danger, where the threat is real rather than imagined. For example, behavioural techniques designed to reduce avoidance may not be appropriate when avoidance is necessary for survival. In such settings, what might be labelled as “symptoms” can instead reflect adaptive responses to persistent risk (25–27). This limitation has led to the development of alternative conceptual frameworks better suited to understanding distress under ongoing trauma exposure.

### Continuous traumatic stress

Continuous traumatic stress (CTS), as an exposure and a psychological response, offers such an alternative conceptual framework. Originally articulated by anti-apartheid mental health practitioners in 1980s South Africa (28) and later formalised by Eagle and Kaminer (26), CTS refers to the psychological impact of living under conditions of ongoing, structurally maintained, and unpredictable threat. Unlike PTSD, CTS symptoms emphasise the psychological impact of present and anticipated trauma exposure rather than the re-experiencing of past events (29). CTS encompasses both exposure to continuous violence, often conceptualised as Type III trauma, including intersected discrimination, prolonged childhood adversities, ongoing intergroup conflict and civil wars, chronic community violence, chronic life-threatening medical conditions, and associated emotional, cognitive, and physiological responses that emerge in such contexts (30).

To operationalise this construct not only as an exposure, but also as a response, Goral et al. (27) developed the Continuous Traumatic Stress Response Scale (CTSR), an 11-item measure assessing symptoms across three proposed dimensions, including fear/helplessness, rage/betrayal, and exhaustion/detachment. Initial validation studies were conducted in Israel, and the scale demonstrated good psychometric properties with high internal consistency (*α* = 0.90) and confirmatory factor analysis indicating an excellent model fit (*χ*^2^ = 49.82, df = 35, *p* = 0.05). Additionally, findings showed CTSR scores and PTSD symptoms were correlated (*r* = 0.720, *p* < 0.001), but only 35–39% of the variance in CTSR subscale scores was shared with PTSD symptoms, supporting the conceptual and empirical distinction of CTS symptoms from PTSD symptoms. Notably, CTS symptom severity was more strongly associated with perceived threat and fear of injury or death than with objective exposure to traumatic events (27).

Subsequent validation studies of the CTSR were conducted in Ukraine, Lithuania, and other conflict-affected populations (20, 31, 32). While these studies generally support the scale’s reliability and validity, they also reveal variation in factor structure, with some adaptations suggesting overlapping dimensions or culturally specific symptom configurations. Such findings raise questions about the cross-cultural stability of CTS symptomatology and highlight the need for alternative analytic approaches that move beyond latent variable models.

Despite the growing recognition of CTS symptomatology as a distinct construct, empirical research examining how CTS symptoms interact with other forms of psychopathology remains limited. Existing studies indicate strong associations between CTS exposure and PTSD symptoms, depression, and anxiety in both Ukrainian and South African populations (32–36). Further, from a network theory perspective, mental disorders are best characterised as direct interactions between different components in a psychopathology network as well as the presence of symptom-symptom connections for accurate diagnoses (37). Moreover, the network approach is particularly useful for estimating and visualising the interrelationships among symptoms at both the group and individual levels (38). However, most research relies on total scale scores and does not examine how specific CTS symptoms may bridge multiple diagnostic domains nor whether such patterns are consistent across cultural contexts.

These gaps are particularly salient in adolescent populations. Adolescence is a developmental period marked by rapid psychological, biological, and social change, rendering young people especially vulnerable to the effects of continuous threat (39, 40). Exposure to war, chronic violence, and instability during adolescence has been consistently linked to elevated PTSD symptoms, depression, and anxiety (8, 19, 31, 41, 42). Yet little is known about how CTS symptoms are structurally embedded within adolescent psychopathology and which specific CTS symptoms exert the strongest cross-domain influence on disorders commonly associated with ongoing adversity.

### Aim and objectives

To address these gaps, this study aims to examine the network structure of CTS symptoms, PTSD, depression, and anxiety symptoms at the item level among adolescents exposed to continuous threat in South Africa and Ukraine. The study addresses four research questions among adolescents exposed to continuous threat:

1. How are CTS symptoms structurally embedded within the PTSD–depression–anxiety symptom network at the item level?
2. Which specific CTS symptoms exert the strongest cross-domain influence on PTSD, depression, and anxiety symptoms, and what type of psychological experiences do these key bridging symptoms represent?
3. To what extent do the CTS symptoms with the strongest cross-domain influence serve as predictors of overall PTSD, depression, and anxiety symptom severity?
4. To what extent are the network structure, global network strength, central CTS symptoms, and bridge symptoms invariant across South African and Ukrainian adolescents?

## Methods

Adolescents aged 12 to 18 years were recruited in South Africa (*n* = 629) through door-to-door sampling and in Ukraine (*n* = 504) via online advertising and a coordinated school-based distribution system. In Ukraine, the survey link was disseminated through regional education authorities, school directors, and class teachers, ensuring broad coverage across schools and communities and enhancing sample representativeness during the war.

In the South African dataset, 600 complete cases were retained for analysis (completion rate = 95.39%). Of the 629 recruited participants, 24 cases (3.82%) were excluded because of incomplete responses, and 5 cases (0.79%) were identified as statistical outliers and excluded, resulting in 29 excluded cases (4.61%) overall. In the Ukrainian dataset, 485 complete cases were retained (completion rate = 96.23%). Of the 504 recruited participants, 15 cases (2.98%) were excluded because of incomplete responses, and 4 cases (0.79%) were identified as statistical outliers and excluded, resulting in 19 excluded cases (3.77%) overall. Thus, the proportion of excluded cases was small in both samples and the exclusions were limited to incomplete responses and a small number of statistical outliers.

Following informed consent and assent procedures, participants completed survey questionnaires using paper-and-pencil (South Africa) and the Qualtrics online platform (Ukraine). The South African-based study was approved by the Health Research Ethics Committee of Stellenbosch University (reference number N24/02/011), the Western Cape Department of Education (reference number 3A65AF1475000018-20240213), and the Psychology Research Ethics Committee of Cambridge University (reference number PRE.2023.014). The Ukrainian-based study was approved by Lesya Ukrainka Volyn National University Ethics Committee (reference number N03-23/04/1017 dated 02/04/2024).

### Measures

The survey questionnaires were translated into the respective local languages (South Africa: Afrikaans, English, Xhosa) and checked for accuracy. Most measures used in the Ukrainian sample underwent cultural adaptation by the Ukrainian Institute of Cognitive Behavioral Therapy (2012) and psychometric validation (32). Participants completed a socio-demographic questionnaire, including age, sex, home language, and standardised measures of PTSD symptoms, depression, anxiety, and the CTSR.^1^ Internal consistency of the study measures was evaluated using Cronbach’s alpha (*α*), providing complementary estimates of scale reliability. All the measures had high reliability in both samples, with alpha scores ranging from 0.84 to 0.96 (see **Table 1**).

**Table 1:** Sample demographic description and scale scores.

Variables	Ukrainian sample		South African sample	
	(*n* = 485)		(*n* = 600)	
Age (years)				
Mean (SD)	15.54 (1.51)		14.94 (1.97)	
Range	12 – 18		12 – 18	

	*n* (%)		*n* (%)	
Sex				
Female	299 (61.65)		221 (36.9)	
Male	184 (37.94)		369 (61.5)	
Other	2 (0.41)		2 (0.3)	
Prefer not to say	–		8 (1.3)	
Previous family psychotrauma (Holodomor)			NA	
Yes	144 (29.69)			
No	341 (70.31)			
Previous family psychotrauma (Deportation to Siberia, repression and other)			NA	
Yes	11 (2.27)			
No	474 (97.73)			
Displaced			NA	
Yes	70 (14.43)			
No	415 (85.57)			

	*M* (SD)	*α*	*M* (SD)	*α*
CPSS-5	22.54 (19.24)	0.96	23.63 (16.49)	0.92
PHQ-9	6.87 (6.29)	0.91	7.35 (6.02)	0.85
GAD-7	5.76 (5.32)	0.93	5.85 (4.97)	0.84
CTSR	6.79 (6.86)	0.91	8.92 (7.13)	0.88

#### CTS response

The CTSR scale (27) measures three distinct factors, including (i) exhaustion/detachment, (ii) range/betrayal, and (iii) fear/helplessness. Participants report the occurrence and severity of experiences (11 items) over the past month on a Likert-type scale ranging from 0 = not at all, to 3 = severe. Higher scores indicate increased CTS symptoms.

#### PTSD

The Child PTSD Symptom Scale for DSM-5 (CPSS-5) (43) is a 20-item modified self-report scale developed for use among children and adolescents. Items are rated on a Likert-type scale ranging from 0 = not at all, to 4 = six or more times a week/almost always. The 20 items are summed to obtain a total symptom severity score (0-10 = minimal; 11-20 = mild; 21-40 = moderate; 42-60 = severe; and 61-80 = very severe).

#### Depression

The Patient Health Questionnaire Modified for Teens (PHQ-9) (44) was adapted from the original PHQ-9 (44, 45) and measures emotional distress, including depression. The nine items are rated on a Likert-type scale ranging from 0 = not at all, to 3 = nearly every day. Cut-off scores indicate mild (5), moderate (10), moderately severe (15), and severe (20) depression (range = 0 to 27).

#### Anxiety

The Generalized Anxiety Disorder scale (GAD-7) (46) measures emotional distress, including anxiety. The seven items are scored on a Likert-type scale ranging from 0 = not at all, to 3 = nearly every day. Higher scores indicate more severe anxiety symptoms.

### Data analysis

Data were analysed using SPSS and R. The use of validated measures, together with the assessment of internal consistency, helped to evaluate the reliability of the symptom measures used in the analyses.

We conducted an item-level network analysis to examine the relationships between CTSR and PTSD, depression, and anxiety symptoms. All CTSR and outcome items (PHQ-9, GAD-7, and CPSS-5) were combined into a single dataset. Within the psychopathology network framework, symptoms were modelled as nodes and their conditional associations as edges. A Gaussian Graphical Model (GGM) with EBICglasso regularisation was estimated using the *estimateNetwork* function in the *bootnet* package, with visualisation conducted using *qgraph*. Although the individual symptom items were measured using ordered Likert-type response categories, they were treated as approximately continuous for the network estimation. The EBICglasso procedure estimates regularised partial correlations between items while controlling for the influence of all other nodes and shrinking small associations toward zero to produce a sparse and interpretable network. Network accuracy and stability were assessed using bootstrap procedures.

An item-level network was estimated, including CPSS-5, PHQ-9, GAD-7, and CTSR scale items. In the network, nodes represent individual items, and edges represent regularised partial correlations controlling for all other items. Nodes were coloured by symptom domain with CPSS-5 = orange; PHQ-9 = blue; GAD-7 = green; and CTSR = pink. Edge thickness and colour intensity indicate the strength of associations.

To identify the most central bridging CTSR items, we selected the top three CTSR items with the highest maximum cross-domain edges, representing the strongest potentially cross-cutting symptom domains for each country sample. These top bridging items were visualised in a horizontal bar plot, with the top three highlighted in red and the remaining CTSR items in orange, with their corresponding edge weights displayed at the end of each bar. The regressions were conducted as a follow-up analysis addressing Research Question 3, which examined whether the CTS symptoms showing the strongest cross-domain influence were associated with overall PTSD, depression, and anxiety symptom severity. Thereafter, multiple linear regression analyses were conducted to examine whether CTSR items identified as bridging symptoms in the network analysis predicted PTSD, depression, and anxiety symptoms, respectively. Specific CTSR items per country included items 3, 10, and 2 for South Africa, and items 4, 8, and 9 for Ukraine. All predictors were entered simultaneously. Standardised regression coefficients (*β*) and explained variance (*R*²) are reported.

At the final stage, we applied the Network Comparison Test (NCT) to examine whether the symptom networks differed significantly between South African and Ukrainian adolescents. NCT is a permutation-based statistical method used to compare symptom networks across independent groups (47). Specifically, it tests differences in overall network structure, individual edge strengths (connections between symptoms), and global network strength (overall connectivity of the network). This approach allowed us to determine whether the organisation of CTSR, PTSD, depression, and anxiety symptoms, and the role of key bridging symptoms, were consistent across the two cultural contexts.

## Results

### Sample characteristics

**Table 1** provides a detailed description of pertinent demographic data of the respective samples. For both samples, scores indicated moderate PTSD symptoms, mild to moderate depression, and mild anxiety.

### Network analysis

The bootstrap analyses indicated that the estimated edge weights were generally stable, with relatively narrow 95% confidence intervals. The case-dropping bootstrap further indicated good stability of the centrality and bridge estimates, with the correlation stability (CS) coefficients exceeding the recommended minimum value of 0.25 and preferably approaching or exceeding 0.50. These findings suggest that the main network and bridge estimates were reasonably robust to sampling variability. A detailed report of all weights above 0.05 is reported in **Supplementary Table S1**.

#### South Africa

Item-level network analysis demonstrated that CTS symptoms served as central bridging nodes linking PTSD, depression, and anxiety symptoms, highlighting the role of CTS symptoms in comorbidity (see **Figure 1**).

**Figure 1:**
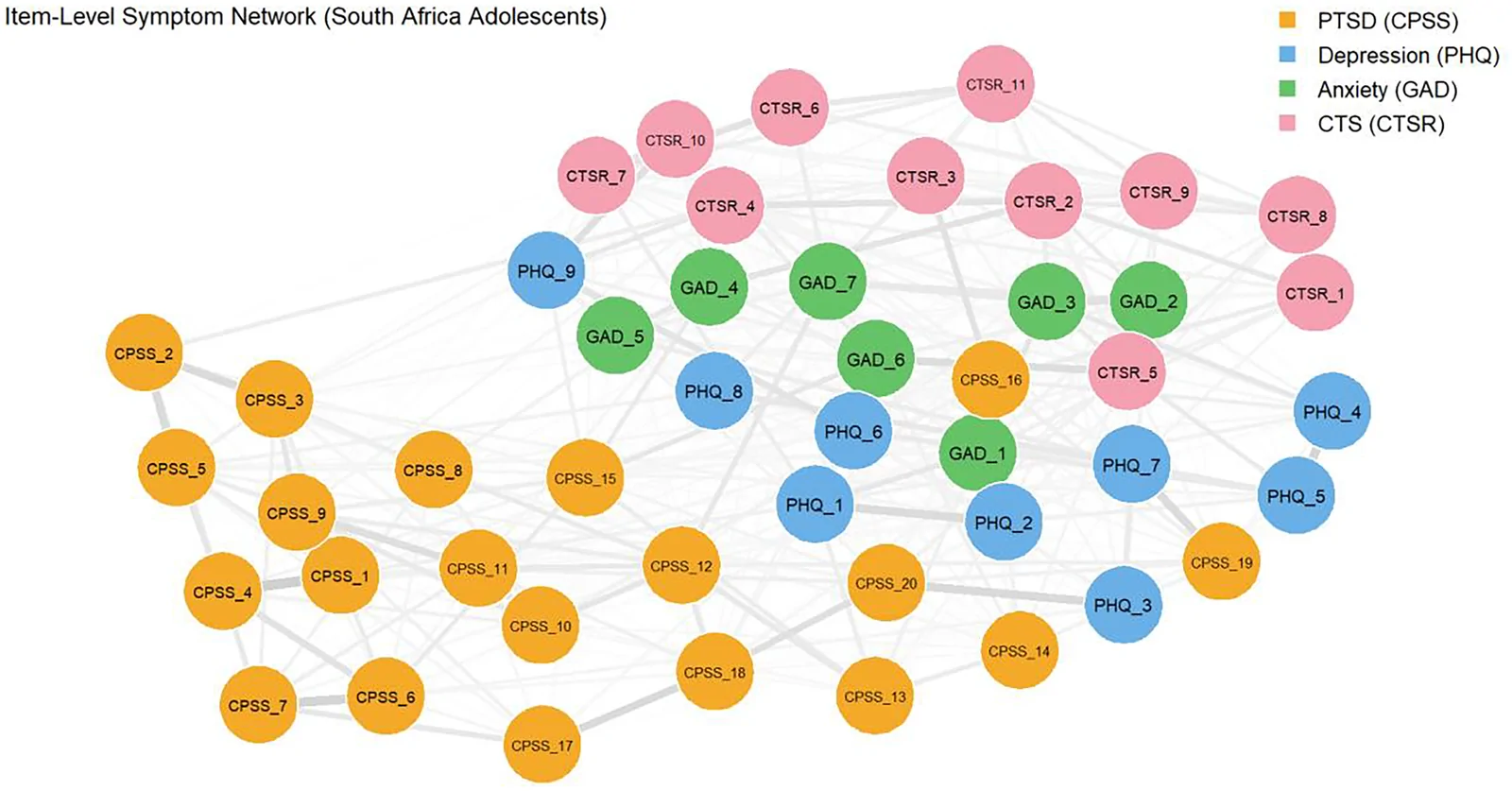
Item-Level continuous traumatic stress-posttraumatic stress disorder-depression-anxiety network (South Africa). CPSS, Child PTSD Symptom Scale for DSM-5; CTSR, Continuous Traumatic Stress Response scale; GAD, Generalized Anxiety Disorder; PHQ, Patient Health Questionnaire. CTSR_2, I feel mentally exhausted; CTSR_3, I feel that my life is in danger; CTSR_4, I feel that my life has no meaning; CTSR_5, I have difficulty controlling my emotions; CTSR_6, I find it hard to trust the people around me; CTSR_7, I feel that no one understands me; CTSR_10, I feel betrayed.

In the South African sample, the strongest associations between CTSR items and symptom domains were generally small to moderate, indicating a diffuse pattern of relationships across anxiety, depression, and PTSD symptoms.

The strongest overall association was between CTSR_5 and GAD_6 (“Becoming easily annoyed or irritable”; weight = 0.164), followed by CTSR_10 and PHQ_9 (“Thoughts that you would be better off dead, or of hurting yourself in some way”; weight = 0.151).

CTSR_3 showed consistent links with CPSS_16 (“Doing things that might hurt yourself”; weight = 0.135) and depressive symptoms, including feeling unusually restless or slowed down (PHQ_8, weight = 0.083; PHQ_9, weight = 0.054), suggesting relevance across both trauma-related and affective symptom clusters. Depressive symptoms were also linked to several CTSR items, including CTSR_4 and PHQ_9 (“Thoughts that you would be better off dead, or of hurting yourself in some way”; weight = 0.107) and CTSR_5 and PHQ_5 (“Feeling tired or having little energy”; weight = 0.098).

Similarly, CTSR_2 was associated with multiple anxiety indicators, including trouble relaxing (GAD_4, weight = 0.133), excessive worry (GAD_2, weight = 0.095; GAD_3, weight = 0.088), irritability, and fear about the future, as well as PTSD symptoms (CPSS_19: “Having trouble paying attention”; weight = 0.051), reflecting cross-domain emotional dysregulation.

Overall, the pattern in the South African sample suggests broad but weakly distributed associations across CTSR items and symptom domains, with anxiety-related outcomes showing the most consistent links, followed by depression and PTSD symptoms.

As indicated in **Figure 2**, CTSR_5 (partial correlation = 0.164), CTSR_10 (partial correlation = 0.151), and CTSR_3 (partial correlation = 0.135) were the strongest bridging symptoms. Specifically, they had the strongest connections to PTSD, depression, and anxiety symptoms in the present South African sample.

**Figure 2:**
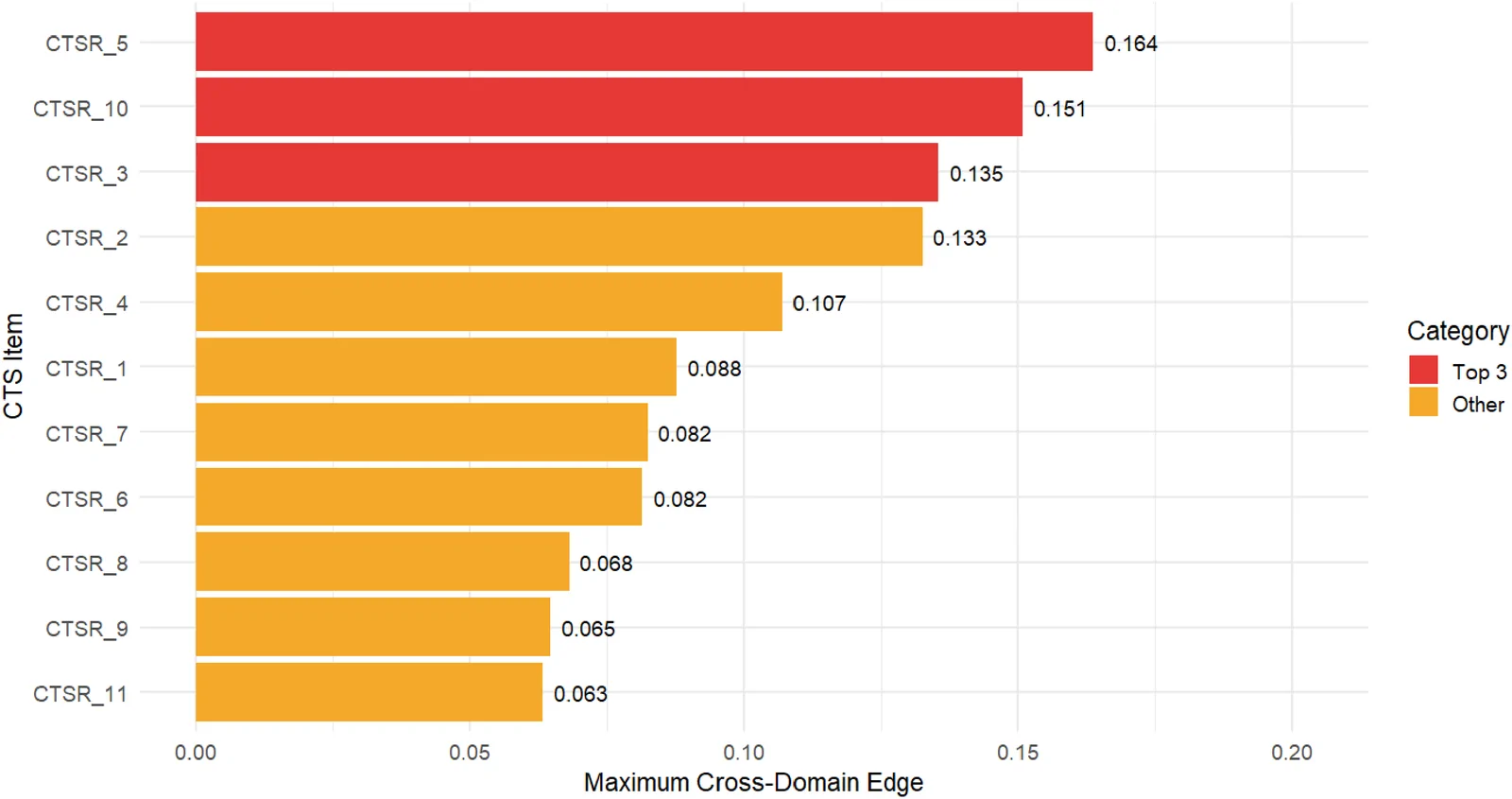
Continuous traumatic stress bridging symptoms and partial correlations (South Africa). CTSR, Continuous Traumatic Stress Response scale. CTSR_3, I feel that my life is in danger; CTSR_5, I have difficulty controlling my emotions; CTSR_10, I feel betrayed.

##### CTSR symptoms as predictors of PTSD, depression, and anxiety symptoms

As indicated in **Table 2**, the three CTSR items were significant positive predictors in all three regression models. Notably, the overall regression model predicting PTSD symptoms was significant (*p* < 0.001), explaining 40.7% of the variance in CPSS-5 scores. Accordingly, the regression model for depressive symptoms was significant (*p* < 0.001), accounting for 50.3% of the variance in PHQ-9 scores. Lastly, the regression model predicting anxiety symptoms was significant (*p* < 0.001), explaining 53.0% of the variance in GAD-7 scores.

**Table 2:** Multiple linear regression predicting posttraumatic stress disorder, depression, and anxiety symptoms (South Africa).

CTSR item	*B*	SE	*B* 95% CI [LL, UPL]	*β*
Predictor	CTS bridging symptoms of PTSD (CPPS-5)			
CTSR_3	2.63	0.70	[1.26, 4.00]	0.14***
CTSR_10	5.62	0.58	[4.48, 6.76]	0.35***
CTSR_5	5.78	0.67	[4.47, 7.09]	0.31***
Model: *R*^2^ = 0.407; Δ*R*^2^ = 0.404; *F* = 139.20***				
Predictor	CTS bridging symptoms of depression (PHQ-9)			
CTSR_3	1.43	0.70	[0.98,1.88]	0.21***
CTSR_10	1.98	0.19	[1.61, 2.35]	0.34***
CTSR_5	2.36	0.22	[1.93, 2.79]	0.35***
Model: *R*^2^ = 0.503; Δ*R*^2^ = 0.500; *F* = 204.90***				
Predictor	CTS bridging symptoms of anxiety (GAD-7)			
CTSR_3	0.91	0.19	[0.54, 1.28]	0.16***
CTSR_10	1.75	0.15	[1.46, 2.04]	0.36***
CTSR_5	2.16	0.18	[1.81, 2.51]	0.39***
Model: *R*^2^ = 0.530; Δ*R*^2^ = 0.528; *F* = 228.80***				

*n* = 612. CPSS, Child PTSD Symptom Scale for DSM-5; CTSR, Continuous Traumatic Stress Response scale; GAD, Generalized Anxiety Disorder; PHQ, Patient Health Questionnaire. Seventeen cases were excluded due to missing data. *B* = unstandardised regression coefficient; *β* = standardised coefficient; SE = standard error. CTSR_3 = I feel that my life is in danger, CTSR_10 = I feel betrayed, and CTSR_5 = I have difficulty controlling my emotions. ***p < 0.001.

#### Ukraine

Item-level analysis of CTSR correlations with PHQ-9, GAD-7, and CPSS-5 scores revealed several prominent bridging patterns (see **Figure 3**).

**Figure 3:**
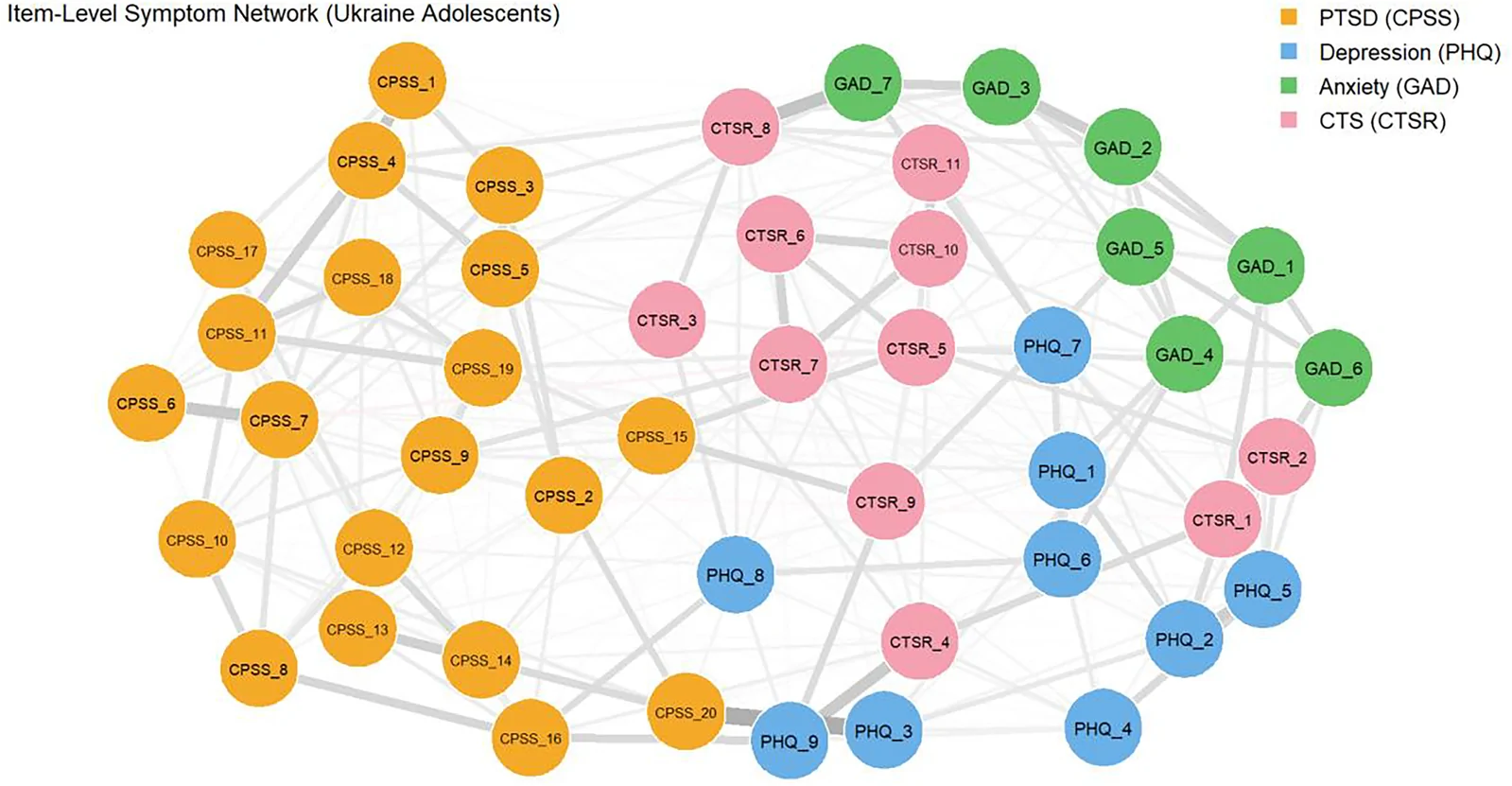
Item-level continuous traumatic stress-posttraumatic stress disorder-depression-anxiety network (Ukraine). CPSS, Child PTSD Symptom Scale for DSM-5; CTSR, Continuous Traumatic Stress Response scale; GAD, Generalized Anxiety Disorder; PHQ, Patient Health Questionnaire. CTSR_2, I feel mentally exhausted; CTSR_4, I feel that my life has no meaning; CTSR_6, I find it hard to trust the people around me; CTSR_8, I have intense feelings of fear or horror; CTSR_9, I have episodes of rage.

In contrast to the South African sample, where anxiety-related associations were most consistently dominant, the Ukrainian sample demonstrated a more evenly distributed and tightly interconnected pattern of associations across anxiety, depression, and PTSD symptoms. The strongest overall association was between CTSR_8 and GAD_7 (“Feeling afraid as if something awful might happen”; weight = 0.288). This was followed by a strong association between CTSR_4 and PHQ_9 (“Thoughts that you would be better off dead, or of hurting yourself in some way”; weight = 0.253).

CTSR_9 showed consistent links across PTSD symptoms, reflecting re-experiencing and hyperarousal (CPSS_15, weight = 0.175), and depression (PHQ_9, weight = 0.170), suggesting that this CTS dimension may represent a broader transdiagnostic stress factor. Similarly, CTSR_2 was associated with both anxiety (GAD_6: “Becoming easily annoyed and irritable”; weight = 0.171) and depression (PHQ_5: “Poor appetite, weight loss or overeating”; weight = 0.118; PHQ_2: “Feeling down, depressed, irritable, or hopeless”, weight = 0.083), indicating overlap across emotional symptom domains.

As indicated in **Figure 4**, CTSR_8 (partial correlation = 0.288), CTSR_4 (partial correlation = 0.253), and CTSR_9 (partial correlation = 0.175) were the strongest bridging symptoms. As such, they had the strongest connections to PTSD, depression, and anxiety symptoms in the Ukrainian sample.

**Figure 4:**
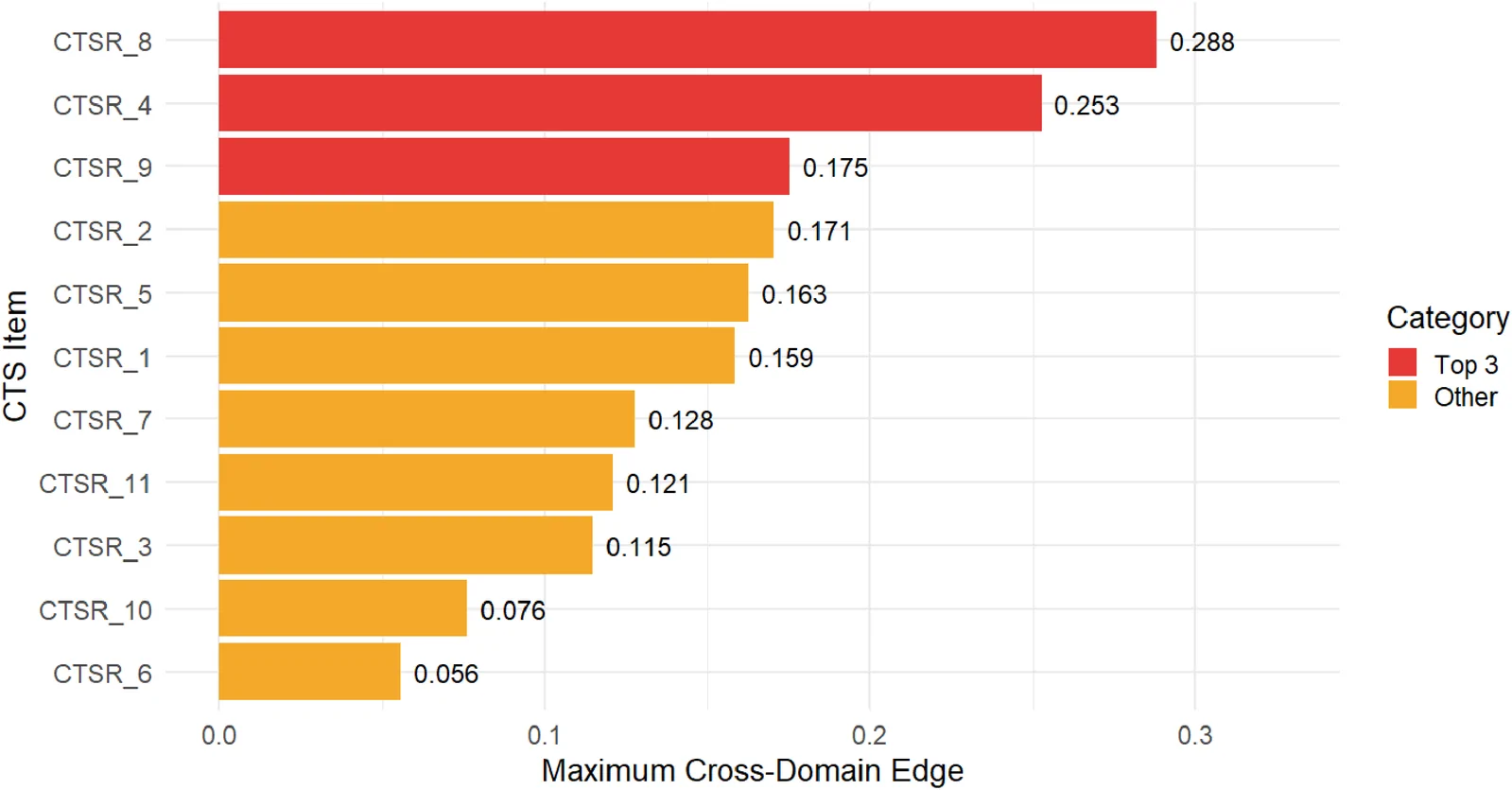
Continuous traumatic stress bridging symptoms and partial correlations (Ukraine). CTSR, Continuous Traumatic Stress Response scale. CTSR_4, I feel that my life has no meaning; CTSR_8, I have intense feelings of fear or horror; CTSR_9, I have episodes of rage.

##### CTSR symptoms as predictors of PTSD, depression, and anxiety symptoms

As indicated in **Table 3**, the three CTSR items were significant positive predictors in all three regression models. Notably, the overall regression model predicting PTSD symptoms was significant (*p* < 0.001), explaining 55.8% of the variance in CPSS-5 scores. Accordingly, the overall regression model predicting depression was significant (*p* < 0.001), explaining 61.5% of the variance in PHQ-9 scores. Lastly, the overall regression model predicting anxiety was significant (*p* < 0.001), explaining 58.8% of the variance in GAD-7 scores.

**Table 3:** Multiple linear regression predicting PTSD symptoms, depression, and anxiety (Ukraine).

CTSR item	*B*	SE	*B* 95% CI [LL, UPL]	*β*
Predictor	CTS bridging symptoms of PTSD (CPPS-5)			
CTSR_4	6.076	0.878	[4.350, 7.801]	0.88***
CTSR_8	10.668	0.978	[8.746, 12.590]	0.98***
CTSR_9	4.845	0.823	[3.228, 6.462]	0.82***
Model: *R*^2^ = 0.558; Δ*R*^2^ = 0.555; *F* = 202.10***				
Predictor	CTS bridging symptoms of depression (PHQ-9)			
CTSR_4	2.705	0.268	[2.179, 3.231]	0.27***
CTSR_8	2.732	0.298	[2.146, 3.318]	0.30***
CTSR_9	1.876	0.251	[1.383, 2.369]	0.25***
Model: *R*^2^ = 0.615; Δ*R*^2^ = 0.613; *F* = 256.00***				
Predictor	CTS bridging symptoms of anxiety (GAD-7)			
CTSR_4	0.863	0.244	[0.384, 1.342]	0.24***
CTSR_8	3.639	0.272	[3.105, 4.173]	0.27***
CTSR_9	1.774	0.229	[1.325, 2.223]	0.23***
Model: *R*^2^ = 0.588; Δ*R*^2^ = 0.585; *F* = 256.00***				

*n* = 485. CPSS, Child PTSD Symptom Scale for DSM-5; CTSR, Continuous Traumatic Stress Response scale; GAD, Generalized Anxiety Disorder; PHQ, Patient Health Questionnaire. *B*, unstandardised regression coefficient; *β*, standardised coefficient; SE, standard error. CTSR_4, I feel that my life has no meaning, CTSR_8, I have intense feelings of fear or horror, and CTSR_9, I have episodes of rage. ***p < 0.001.

### Cross-country network comparison

The NCT revealed a significant difference in overall network structure between Ukraine (*n* = 485) and South Africa (*n* = 558), *M* = 0.288, *p* < 0.001, based on 1,000 permutations. The global strength differed significantly between the two networks (S = 0.899, *p* = 0.003), indicating higher overall connectivity in the Ukrainian network (GS = 23.067) compared to the South African network (GS = 22.1677). Further inspection of edge invariance showed that 114 edges differed significantly at the uncorrected level, with 13 edges remaining significant after Bonferroni–Holm correction (see **Figure 5**).

**Figure 5:**
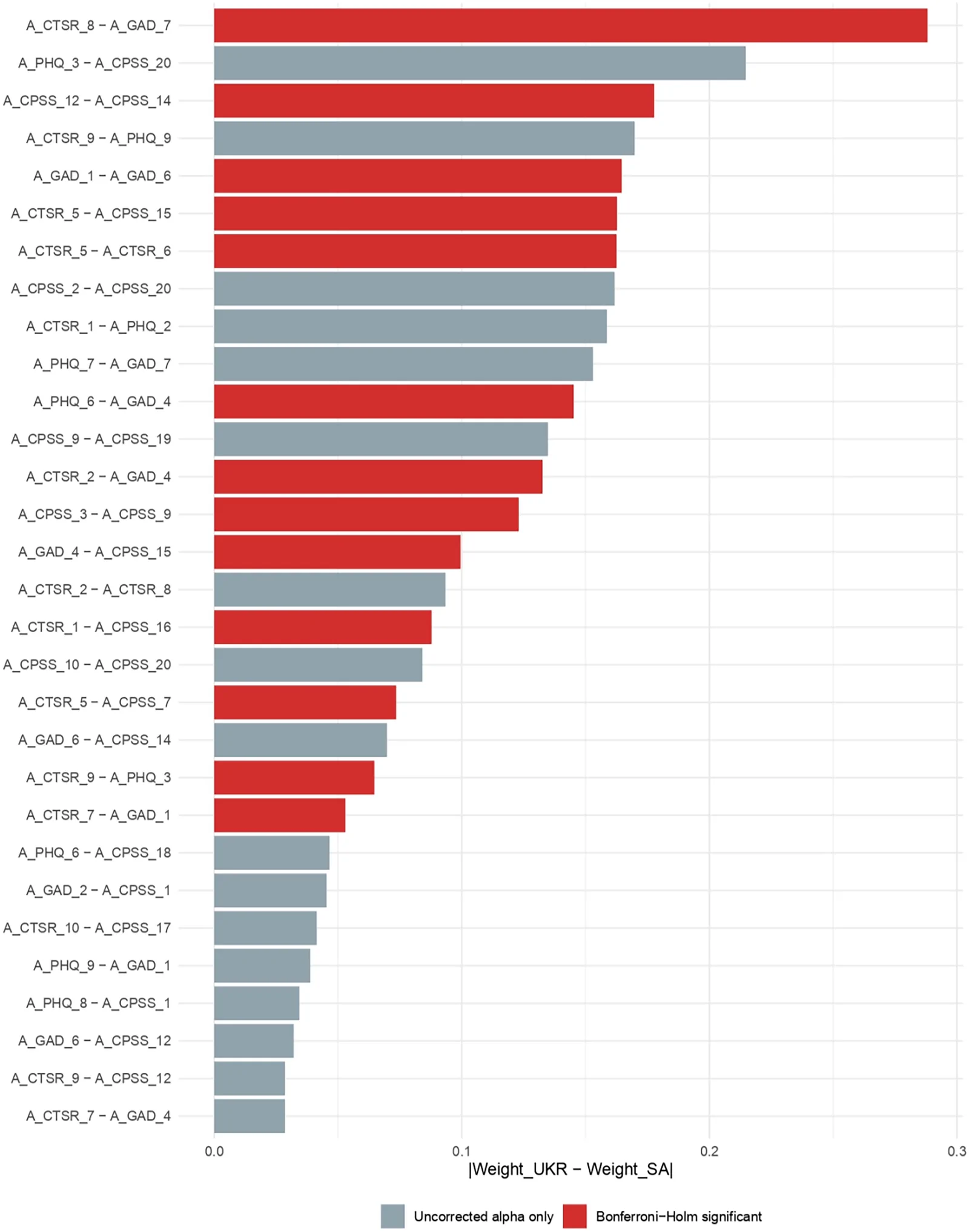
Significant edge differences between Ukraine and South Africa (*post-hoc* edge tests). CPSS, Child PTSD Symptom Scale for DSM-5; CTSR, Continuous Traumatic Stress Response scale; GAD, Generalized Anxiety Disorder; PHQ, Patient Health Questionnaire. CTSR_5, I have difficulty controlling my emotions; CTSR_7, I feel that no one understands me; CTSR_8, I have intense feelings of fear or horror; CTSR_9, I have episodes of rage; CTSR_10, I feel betrayed.

Of specific interest were the item-specific variations in PTSD symptoms, including (i) CTSR_10 and CPSS_17 (“Being very careful or on the lookout for danger”), which highlighted the variation in the role of interpersonal mistrust between countries; and (ii) CTSR_5 and CPSS_6 (“Trying not to think about it or have feelings about it”), CPSS_7 (“Trying to stay away from anything that reminds you of what happened”), and CPSS_15 (“Getting angry easily”), which indicated that emotion dysregulation operated differently across contexts.

In terms of depression, item-specific variations, including CTSR_9 and PHQ_3 (“Trouble falling asleep, staying asleep, or sleeping too much”) and PHQ_9 (“Thoughts that you would be better off dead or hurting yourself in some way”), indicated that rage may differentially coexist with emotional withdrawal, sleep disruption, and suicidal ideation between the two countries.

Lastly, item-specific variation in anxiety symptoms included CTSR_8 and GAD_7 (“Feeling afraid as if something awful might happen”), which highlighted the variation in how fear was translated into anticipatory anxiety and future-oriented threat between countries; and CTSR_7 and GAD_1 (“Feeling nervous, anxious, or on edge”) and GAD_4 (“Trouble relaxing”), which indicated that perceived social isolation differentially contributed to persistent anxiety and difficulty relaxing across contexts.

## Discussion

### CTS symptoms are structurally embedded with the PTSD-depression-anxiety symptom network at the item level

Item-level analyses across both samples demonstrated that CTS symptoms (including motivational depletion and mental exhaustion, emotional dysregulation, persistent threat-related affect, episodes of rage, and social alienation) were not peripheral stress indicators but were structurally embedded as central bridging nodes linking PTSD (including hyperarousal, re-experiencing, and risk-taking or self-harming behaviours), depression (including anhedonia, low energy, hopelessness, sleep disturbance, and suicidal ideation), and anxiety symptom domains (including excessive worry, nervous arousal, irritability, and difficulty relaxing); thereby contributing to their comorbidity. In both samples, CTSR items were consistently connected to symptom domains that are typically examined separately, supporting the transdiagnostic approach whereby shared processes causing symptoms of multiple mental disorders across categories of disorders explain comorbidities (48). Further, the convergence of core CTS symptoms across both samples suggests that CTS is structurally integrated within trauma-related symptom networks.

Taken together, these pooled findings support the potential conceptualisation of CTS as a putatively transdiagnostic process that disrupts emotion regulation, threat processing, and social connectedness, thereby underpinning multi-domain psychopathology in adolescents exposed to ongoing threat. Thus, in this context, trauma-related psychopathology may be organised around a unified stress-response system rather than discrete diagnostic categories. Accordingly, Borsboom (37) posits that, in cases where specific symptoms are embedded as central bridgning nodes, if one symptom is active (e.g., the key bridging CTS symptoms), it is more likely than not that the other symptoms (e.g. relevant PTSD, depression, and anxiety symptoms) will also be active. This is further supported by a recent study suggesting that trauma-focused interventions may need to be adapted for contexts of chronic stress and continuous trauma by targeting shared processes rather than disorder-specific symptoms (49). However, further research is needed to confirm the potential conceptualisation of CTS as a transdiagnostic process.

### CTS symptoms with the strongest cross-domain influence and as predictors of PTSD, depression, and anxiety symptoms

Although symptom-level analyses indicated a more anxiety-dominated pattern in the South African sample and a more evenly distributed, transdiagnostic pattern in the Ukrainian sample, the regression findings suggest a convergent structure across contexts. Thus, despite contextual variation in the expression of symptom associations, the underlying structural interaction of CTS remained stable and consistent across trauma-related domains. These findings demonstrate the multifaceted psychological burden of living in conditions of CTS, and support the conceptualisation of the contributory influence of CTS to multi-domain psychopathology. In the South African sample, CTS symptoms showed the strongest explanatory contribution to anxiety, while in the Ukrainian sample, the strongest contribution was observed for depression, suggesting contextual differences.

Specifically, emotional dysregulation, rage, and betrayal (CTSR_5: strongest; CTSR_10: second strongest) exerted the strongest cross-domain influence, while CTSR_9 showed the third strongest cross-domain influence in the Ukrainian sample. This captures dysregulated anger and emotional reactivity as a natural response to prolonged stress, injustice, and perceived powerlessness. Rage, especially, is consistent with anger symptoms observed across PTSD and anxiety symptoms. Prior research indicated that PTSD symptom severity decreased adolescents’ willingness to forgive and heightened their desire for revenge (50). Combined with the strong cross-domain influence of rage and betrayal, this finding highlights the importance of early interventions to mitigate the long-term effects of CTS and deter the cycle of violence (36). This is especially true within the South African context, where turf wars between rival gangs and a propensity for mob justice (vigilantism) are rife (51, 52).

Fear and hopelessness showed a strong cross-domain influence in both the South African (CTSR_3: third strongest) and Ukrainian (CTSR_8: strongest) samples, representing the persistent activation of threat-related affect and closely linking CTS symptoms with PTSD-related fear and anxiety symptoms. Notably, unlike the conventional PTSD paradigm, these feelings of fear and hopelessness were related to ongoing threat rather than episodic reactions. Interestingly, fear and hopelessness had the strongest cross-domain influence in the Ukrainian sample, while it was only the third strongest in the South African sample, possibly reflecting the different contexts. Specifically, Ukrainian adolescents likely recall a time when they lived without fear and hopelessness before the full-scale invasion, whereas South African adolescents likely never knew conditions without CTS. This difference in baseline exposure may partly explain the stronger psychological response to CTS exposure among Ukrainian adolescents.

Lastly, exhaustion and detachment (CTSR_4) had the second strongest cross-domain influence among the Ukrainian sample. Specifically, adolescents’ sense that their “life has no meaning” reflects the profound existential disruption since their meaning systems are repeatedly challenged by the ongoing war-related threats and loss. It also captures disruption of future orientation and hopelessness, which are commonly observed in populations exposed to chronic war-related stress (53, 54) and are closely aligned with depressive symptomatology.

### Between-country invariance in the network structure, central CTS symptoms, and bridge symptoms

#### CTSR and CPSS-5

Feelings of betrayal appeared to be more strongly embedded in threat-monitoring processes, suggesting that relational insecurity differentially contributed to sustained hyperarousal. This may reflect differences in the social ecology of threat, where distrust of others becomes a central mechanism maintaining trauma-related arousal. Indeed, previous research shows that trauma-related betrayal often extends beyond family relationships to a broader loss of trust in social institutions such as schools, religious communities, political systems, and leadership structures (55). Given that CTSR_10 was the strongest predictor of PTSD symptoms in the South African sample, this finding likely applies to the South African sample, where the apartheid legacy of structural inequality (including poor schooling), impoverishment, and a corrupt government remains, over 30 years after the end of apartheid. Further, in the context of ongoing threat and war, young people may lose confidence in authority figures, communities, and even their own country, which can intensify moral disorientation, psychological distress, and both mental and physical health difficulties (53, 55–58).

Additional differences involving emotion regulation and PTSD symptoms of avoidance and anger further indicate that emotion dysregulation operates differently across contexts as a pathway linking CTS to both internalising and trauma-related symptoms. With CTSR_5 being the second strongest predictor of PTSD symptoms in the South African sample, this finding reiterates the necessity for early interventions in emotion regulation, including dealing with feelings of anger and betrayal, to prevent the cycle of violence.

#### CTSR and PHQ-9

Another important set of differences concerns episodes of rage, which showed stronger associations with depressive symptoms such as sleep disturbance and suicidal ideation in one country compared to the other. Rather than representing only externalised anger, rage may coexist with sleep disruption and suicidal ideation, suggesting a pattern of psychological overload in which anger and hopelessness are closely intertwined (59, 60). This may be particularly important among the Ukrainian adolescents, where CTSR_9 was a strong predictor of depression symptoms. Specifically, in terms of sleep disturbances, the South African sample, who grew up in conditions of CTS, may have adapted to the sounds associated with violence (e.g., gunshots), which is not the case for the Ukrainian sample. Sleep disturbances predict suicidal attempts (61) and ideation (62) and feelings of rage are linked to impulsive behaviours, including suicidality (63); therefore, interventions to improve the sleep quality of Ukrainian adolescents are imperative.

#### CTSR and GAD-7

Feelings of fear and horror showed a stronger connection with the anticipation that something awful might happen in one country compared to the other. With CTRS_8 being the strongest predictor of anxiety among Ukrainian adolescents, this would suggest that fear was more strongly translated into anticipatory anxiety and future-oriented threat perception among Ukrainian adolescents. Thus, the subjective experience of ongoing danger, rather than exposure alone, may likely be a stronger driver of psychological distress in adolescents living under chronic threat. Indeed, recent findings showed that CTS symptom severity was more strongly associated with perceived threat and fear than with objective exposure to traumatic events (27). This could potentially be explained by the different context where the ongoing threat is relatively *new* versus a context where the ongoing threat has been normalised to some extent.

Similarly, feeling misunderstood by others was differentially associated with feeling nervous/anxious/on edge or having trouble relaxing. This may indicate that interpersonal disconnection may more strongly contribute to persistent anxiety and difficulty relaxing in one country compared to the other. This invariance may be explained by the overall more collectivistic culture of the South African sample population (64) compared to the slightly more individualistic culture of Ukraine (65, 66). Further, recent evidence highlighted that positive parenting, secure attachment, and supportive school or institutional climates are important protective factors against CTS in young people (67). These protective relational environments may reduce anxiety by strengthening adolescents’ sense of safety and social belonging despite ongoing external threat.

### Implications

The NCT demonstrated important differences between the South African and Ukrainian symptom networks. These cross-network links suggest that CTS could potentially operate as a transdiagnostic mechanism through which ongoing threat contributes to broader psychological distress. The results also support the notion that CTS is not simply an extension of PTSD but a distinct process caused by chronic threat and relational instability. Therefore, trauma-informed interventions should target not only PTSD symptoms but also emotion dysregulation, loss of meaning, betrayal, and chronic anticipatory fear. As the most prominent bridging symptoms, it is plausible that targeting these specific CTS symptoms may assist in reducing comorbid distress. Additionally, in conditions of CTS, healthcare providers may consider adjusting conventional PTSD-focused treatments (such as prolonged exposure therapy (68, 69)). For example, given the ongoing exposure, it may be beneficial to place a stronger focus on emotion regulation as opposed to desensitisation (since the threat is ongoing and unavoidable). Further, augmenting PTSD-focused treatments with emotion regulation therapy (70), meaning-centred psychotherapy (71), existential therapy (72), and attachment-based therapies (e.g., emotionally focused therapy) (73) could serve to better address the bridging symptoms of emotion dysregulation, loss of meaning, and feelings of betrayal (especially institutional betrayal, which is prevalent in conditions of CTS (74, 75)).

Further, these findings suggest that CTS does not function at the same level across settings. Instead, it interacts with PTSD, depression, and anxiety symptoms through context-dependent pathways, with interpersonal disconnection, betrayal, fear, and emotion dysregulation playing different bridging roles in South African and Ukrainian adolescents. This supports the view that CTS is a context-sensitive system, where socio-political and environmental conditions shape how distress is organised and maintained across symptom networks (76). Importantly, the Ukrainian network appeared to be more densely interconnected, suggesting stronger mutual reinforcement among symptoms. This may indicate a more tightly connected system, in which activation of one symptom is more likely to spread to others, potentially contributing to greater overall psychological burden under CTS. This greater symptom interconnectivity observed in the Ukrainian network may reflect the detrimental effect of war-related trauma, where adolescents have had less opportunity to adapt to persistent threat conditions compared to contexts of chronic community violence. In contrast, in South Africa, longer-term exposure to violence may have contributed to partial adaptation to ongoing threat, resulting in a somewhat less densely interconnected symptom structure. However, this interpretation remains understudied and should be further investigated in longitudinal studies to better understand how different forms of CTS may be shaped in psychopathology or adaptation.

Overall, the findings suggest that network models may provide a useful framework for understanding psychopathology as a system of interacting symptoms. By identifying relationships among symptoms, network analysis may help clarify influential symptoms and pathways through which CTS-related difficulties reinforce one another and contribute to comorbidity. This complements traditional latent-variable approaches by highlighting symptom-level interactions that may otherwise be obscured.

### Limitations and future recommendations

The study relied on self-report cross-sectional data. As such, the potential impact of response bias should be considered. Longitudinal studies are also needed to examine the directionality of individual symptoms and their relationship with other symptoms and domains over time.

Another limitation concerns the availability and comparability of sociodemographic information across the two samples, as well as differences in recruitment procedures. In South Africa, participants were recruited through door-to-door approaches, whereas in Ukraine, recruitment was conducted primarily through online advertising, and coordinated school-based distribution. Although the comparison between South Africa and Ukraine provides an interesting cross-cultural perspective, the samples differed in several potentially relevant characteristics, including sex distribution, recruitment procedures, sociocultural context, and type of traumatic exposure. In addition, information on other sociodemographic and individual characteristics, such as ethnicity and socioeconomic status was limited. These differences may be associated with variation in network structure independently of CTS and therefore complicate the interpretation of cross-country differences. Future studies should provide more comprehensive information on sample characteristics and, where possible, account for these factors when comparing populations across different sociocultural contexts.

Another limitation is that the regression analyses were exploratory and associative rather than independent causal tests. Because the CTS symptoms were selected from the same dataset based on their network characteristics, the findings should not be interpreted as evidence of independent predictive validity or causality. The present study focused exclusively on psychopathology symptoms and did not examine potentially protective factors, which may buffer the effects of continuous threat and influence the structure and dynamics of psychopathology symptom networks.

Lastly, the NCT indicated significant differences in the overall network structure between the two countries, yet the output did not clearly delineate which specific pattern was attributable to each country. While this could partially be deduced using the strongest cross-domain and predictor items, further research is needed to clarify these between-sample differences.

## Conclusion

CTS symptoms could potentially operate as a cross-cutting process contributing to shared symptom expression across PTSD, depressive, and anxiety disorders in adolescents exposed to ongoing violence. Interventions targeting core CTS bridge symptoms, particularly life threat, betrayal, emotion dysregulation, exhaustion, and social alienation, may reduce comorbidity and improve mental health outcomes in chronically threatened youth across diverse settings.

## Data Availability

The raw data is available upon reasonable request and with a data transfer agreement in place. For the South African data contact Soraya Seedat (sseedat@sun.ac.za) and for the Ukraine data, contact Larysa Zasiekina (l.zasiekina@exeter.ac.uk).

## Footnotes

1 A formal sample comparison to identify covariates (e.g., sex) was not conducted, as network analysis does not rely on covariate adjustment in the same manner as regression-based models. Network models focus on estimating conditional dependencies among variables.
