# Supplementary material for: Network analysis of trauma-related mental health comorbidities under continuous traumatic stress: a comparative study of South African and Ukrainian adolescents
Source: Front Psychiatry. 2026 Oct 13;17:1888773. doi: 10.3389/fpsyt.2026.1888773 (PMC13711263; doi:10.3389/fpsyt.2026.1888773)
Supplement: Supplementary file 1 [file Table1.docx]

**Table S1.** Pair-wise CTSR cross-domain edges with weights 0.05 and above, estimated for Ukrainian and South African samples

| **Ukrainian Sample (n = 485)** | | | **South African Sample (n = 600)** | | |
| --- | --- | --- | --- | --- | --- |
| **CTSR Item** | **Connected To** | **Weight** | **CTSR Item** | **Connected To** | **Weight** |
| CTSR_8 | GAD_7 | 0.288 | CTSR_5 | GAD_6 | 0.164 |
| CTSR_4 | PHQ_9 | 0.253 | CTSR_10 | PHQ_9 | 0.151 |
| CTSR_9 | CPSS_15 | 0.175 | CTSR_3 | CPSS_16 | 0.135 |
| CTSR_2 | GAD_6 | 0.171 | CTSR_2 | GAD_4 | 0.133 |
| CTSR_9 | PHQ_9 | 0.170 | CTSR_4 | PHQ_9 | 0.107 |
| CTSR_5 | CPSS_15 | 0.163 | CTSR_5 | PHQ_5 | 0.098 |
| CTSR_1 | PHQ_2 | 0.159 | CTSR_5 | GAD_3 | 0.098 |
| CTSR_1 | GAD_1 | 0.148 | CTSR_2 | GAD_2 | 0.095 |
| CTSR_9 | GAD_5 | 0.139 | CTSR_2 | GAD_3 | 0.088 |
| CTSR_7 | CPSS_9 | 0.128 | CTSR_1 | CPSS_16 | 0.088 |
| CTSR_11 | PHQ_7 | 0.121 | CTSR_3 | PHQ_8 | 0.083 |
| CTSR_2 | PHQ_5 | 0.118 | CTSR_7 | PHQ_1 | 0.082 |
| CTSR_5 | GAD_6 | 0.116 | CTSR_10 | GAD_6 | 0.082 |
| CTSR_3 | PHQ_8 | 0.115 | CTSR_4 | CPSS_2 | 0.082 |
| CTSR_8 | GAD_2 | 0.103 | CTSR_6 | GAD_7 | 0.082 |
| CTSR_8 | CPSS_3 | 0.099 | CTSR_1 | GAD_1 | 0.071 |
| CTSR_2 | PHQ_2 | 0.083 | CTSR_10 | GAD_7 | 0.071 |
| CTSR_4 | PHQ_4 | 0.080 | CTSR_8 | CPSS_20 | 0.068 |
| CTSR_3 | CPSS_5 | 0.079 | CTSR_9 | PHQ_3 | 0.065 |
| CTSR_10 | GAD_4 | 0.076 | CTSR_11 | GAD_7 | 0.063 |
| CTSR_5 | CPSS_7 | -0.074 | CTSR_3 | GAD_2 | 0.062 |
| CTSR_4 | CPSS_16 | 0.072 | CTSR_8 | GAD_2 | 0.057 |
| CTSR_1 | PHQ_1 | 0.072 | CTSR_1 | PHQ_7 | 0.056 |
| CTSR_8 | PHQ_8 | 0.072 | CTSR_7 | GAD_3 | 0.056 |
| CTSR_2 | PHQ_7 | 0.070 | CTSR_10 | GAD_4 | 0.055 |
| CTSR_11 | PHQ_8 | 0.062 | CTSR_9 | PHQ_2 | 0.054 |
| CTSR_5 | CPSS_18 | 0.062 | CTSR_3 | PHQ_9 | 0.054 |
| CTSR_1 | PHQ_7 | 0.059 | CTSR_10 | PHQ_7 | 0.054 |
| CTSR_5 | GAD_2 | 0.057 | CTSR_5 | GAD_4 | 0.054 |
| CTSR_6 | CPSS_17 | 0.056 | CTSR_8 | PHQ_2 | 0.053 |
| CTSR_8 | GAD_5 | 0.054 | CTSR_7 | GAD_1 | 0.053 |
| CTSR_4 | CPSS_14 | 0.054 | CTSR_5 | CPSS_18 | 0.051 |
| CTSR_7 | PHQ_6 | 0.053 | CTSR_2 | CPSS_19 | 0.051 |
| CTSR_7 | PHQ_8 | 0.051 | CTSR_5 | CPSS_11 | 0.050 |
| CTSR_9 | PHQ_2 | 0.050 |  |  |  |

Note. Weights are rounded to three decimal places. Samples sorted independently by descending weight.
